# A Rare Case of Dislocation

**Published:** 1887-09

**Authors:** C. M. Harrison

**Affiliations:** Tulip, Fannin County, Texas


### A RARE CASE OF DISLOCATION.

*By C. M. Harrison, M. D., Tulip, Fannin County, Texas.*

For Daniel's Texas Medical Journal.

This case is reported because of its rarity. Was called March 29, 1887, by Mr. T., to see his baby, about four weeks old, suffering from a swollen and inflamatory condition of a hand and forearm, together with some febrile disturbance. The swelling was so great that the real condition could not be ascertained. Local treatment consisted of emolient and anodyne applications, while other remedies in common use were prescribed for the febrile condition. April 3, convulsions supervened. The swelling by this time had become sufficiently reduced to reveal a backward dislocation of the carpus. Dr. T., who saw the child with me, and who also delivered it, said there was nothing abnormal at first, and concurred in diagnosis. As to cause of dislocation, I cannot say. Not being congenital, having no convulsions till afterward, parents ignorant of cause, it can only be conjectural. While carpal dislocations are not very rare, carpal dislocations in infants less than a month old are seldom seen on record.

---

"A DEAD ISSUE;" a still born child.

